# From a Low-Cost Air Quality Sensor Network to Decision Support Services: Steps towards Data Calibration and Service Development

**DOI:** 10.3390/s21093190

**Published:** 2021-05-05

**Authors:** Tiago Veiga, Arne Munch-Ellingsen, Christoforos Papastergiopoulos, Dimitrios Tzovaras, Ilias Kalamaras, Kerstin Bach, Konstantinos Votis, Sigmund Akselsen

**Affiliations:** 1 Department of Computer Science, Norwegian University of Science and Technology, 7034 Trondheim, Norway; kerstin.bach@ntnu.no; 2 Telenor Research, 1360 Fornebu, Norway; arne.munch-ellingsen@telenor.com (A.M.-E.); sigmund.akselsen@telenor.com (S.A.); 3 Centre for Research and Technology Hellas, Information Technology Institute, 57001 Thermi, Thessaloniki, Greece; papasterc@iti.gr (C.P.); dimitrios.tzovaras@iti.gr (D.T.); kalamar@iti.gr (I.K.); kvotis@iti.gr (K.V.)

**Keywords:** air quality, low-cost sensors, sensor calibration, warning systems, data visualization

## Abstract

Air pollution is a widespread problem due to its impact on both humans and the environment. Providing decision makers with artificial intelligence based solutions requires to monitor the ambient air quality accurately and in a timely manner, as AI models highly depend on the underlying data used to justify the predictions. Unfortunately, in urban contexts, the hyper-locality of air quality, varying from street to street, makes it difficult to monitor using high-end sensors, as the cost of the amount of sensors needed for such local measurements is too high. In addition, development of pollution dispersion models is challenging. The deployment of a low-cost sensor network allows a more dense cover of a region but at the cost of noisier sensing. This paper describes the development and deployment of a low-cost sensor network, discussing its challenges and applications, and is highly motivated by talks with the local municipality and the exploration of new technologies to improve air quality related services. However, before using data from these sources, calibration procedures are needed to ensure that the quality of the data is at a good level. We describe our steps towards developing calibration models and how they benefit the applications identified as important in the talks with the municipality.

## 1. Introduction

Good air quality in urban areas is essential for human well-being. Since 2008, when the European Union released the Ambient Air Quality EU Directive 2008/50/EC that establishes health-based standards and objectives for pollutants present in the air, the assessment of outdoor air quality has been focused on by municipalities. Assessing and forecasting air quality is a crucial part of the strategies deployed in order to take measures to reduce air pollution and avoid poor air quality for citizens [1]. This highlights the importance of accurate air quality monitoring, which is typically achieved through the deployment of air quality sensor networks. Moreover, air quality affecting the individual depends on local phenomena such as weather, wind, the layout of the city, and pollution sources, which makes the topic interesting for a network of sensors deployed in a city with the goal to collect data and use it for prediction models [2,3,4]. There is a balance to be made when deploying such networks, concerning the coverage of a region against deployment costs. A bulky, industrial sensor type is more accurate but more expensive, both in deployment and in maintenance, while lower-cost sensors reduce by a large margin the deployment costs but at the cost of poorer data quality. However, the research community is working towards methods to improve the quality of data incoming from low-cost sensors, such that they meet minimal requirements for a public evaluation on air quality levels [5]. Therefore, the establishment of low-cost sensor networks can contribute to real-time measurements of the air quality but also allow for using machine learning methods to ensure data quality and predict air quality for the next 24 to 48 h [6].

This paper is motivated by collaboration between universities and public and private entities, and builds on challenges identified by the municipality in its efforts to develop strategies to avoid poor air quality. We explore the feasibility of using a network of low-cost sensors for services such as air quality monitoring and forecasting in a city or decision-making support to decision makers in the municipality. In this context, a first challenge to address is the evaluation of such sensor networks when comparing with air quality data from industrial sensors (in the rest of the document, industrial sensors will be termed as reference sensors) being part of the official pollution measuring network administered by the Norwegian Environmental Agency (NEA). Therefore, we address the challenges of sensor calibration to improve the data quality, which is essential to guarantee that data from low-cost sensors are meaningful and can be trusted by the end users. Sensor calibration can be defined as transforming the measurements from sensors in a way that the calibrated measurements follow closely those from reference instruments, which are typically more expensive and, thus, of better quality [7]. With low-cost sensors it is particularly interesting to investigate calibration procedures with machine leaning methods [8], given the few physical parameters that can be tuned in this kind of sensors and the non-linear influences that external features, such as weather, have on the measurements. Moreover, machine learning based calibration has the advantage of reducing the effort in the calibration process [8] and allows for remote calibration over a communication network. Finally, with good calibration procedures it is possible to trust data to be useful for services to end user. This paper presents visualizations and possible applications that utilize the air quality sensor network.

The paper is organized as follows. Section 2 introduces the context and motivation for the deployment of a network of low-cost air quality sensors, along details about the deployment. In Section 3 we discuss the issue of data calibration with different approaches and evaluate the effect of the calibration procedure in the dataset. Section 4 presents some applications that benefit from using calibrated data and are motivated by the services in which the local municipality showed most interest.

## 2. Background and Motivation

### 2.1. Air Quality Data Pipeline

**Figure 1:**
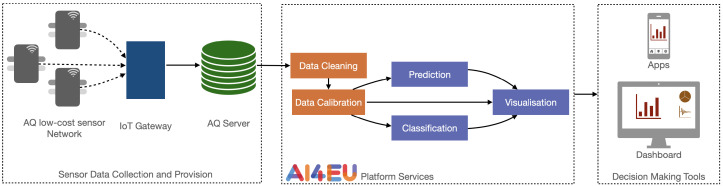
Overview of data collection, process and application pipeline.

The Norwegian University of Science and Technology (NTNU), Telenor and the Information Technologies Institute, Centre for Research and Technology Hellas (ITI-CERTH) have collaborated with the municipality of Trondheim (Norway) since August 2018 on the exploration of options for improved air quality services based on new technologies within the context of artificial intelligence (AI) and Internet of Things (IoT). This collaboration is referred to as the AI4IoT (https://research.idi.ntnu.no/ai4eu/, accessed on 3 May 2021) pilot on air quality monitoring and ran within the framework of the AI4EU project, whose objective is to build a European AI on-demand platform. The pilot focused on technologies for data capture, advanced analyses and visualizations and has addressed the different parts of a value chain for a complete solution (see Figure 1). The services and data provided for this pilot will be part of the European AI on-demand platform to showcase its use.

Figure 1 provides an overview of how data are collected and processed in the air quality pilot. It consists of three components: first the low-cost sensor network, which collects and regularly sends data via an IoT gateway to an air quality server (AQ Server) that stores and adjusts the data based on the lab calibration parameters. The AQ Server is the data source for various AI4EU platform services. Those services can be standalone services or linked in a pipeline. They access data from the AQ Server and can then process them to create prediction or classification models and their visualizations. In this paper, we focus on supervised learning but the pipeline is general enough to accommodate any other type of model. Those components can then be accessed by decision-making tools for providing air quality information to citizens or decision makers.

### 2.2. Application Domain: Air Quality Pilot in Trondheim

The municipality of Trondheim (Norway) has experienced periods of poor air quality, often as a result of high particle dust levels stemming from traffic. Air quality standards are defined in directives issued by the European Union on ambient air quality and cleaner air for Europe (2008/50/EC and 2004/107/EU). The specified air quality standards are based on existing research on the health effects of exposure to pollution components. Norway has agreed to follow these regulations, which for PM10 means that the threshold level of 50 μg/m^3^ (average over 24 h) should not be exceeded more than 30 times a year. In 2013, the Norwegian Environmental Agency ordered Trondheim municipality, Trøndelag county and the Norwegian Road Authorities (in their roles as pollution authorities and road owners) to collaborate on how to improve the situation, in the follow up of Norway having been taken to court over high pollution levels (https://www.eftasurv.int/newsroom/updates/internal-market-norway-be-brought-court-over-air-pollution, accessed on 3 May 2021). Actions were taken in terms of regulations (e.g., related to traffic), but the municipality wants to further extend these measures with the use of intelligent decision support systems. As new research suggests that there are indications of health effects with lower exposure levels the Norwegian Health and local authorities have set even more ambitious targets. According to the annual report on air quality in the city of Trondheim [9]: “The air quality in Trondheim is satisfactory most of the year, but the particulate matter fraction PM_10_ is a problem from October to May due to the use of studded tires on ice-free roads. Particulate matter from transport to and from construction sites, landfills and quarries is also a problem locally. Nitrogen dioxide (NO_2_) from diesel engines is also a problem during winter. This problem is not related to peak values, but to annual mean concentration. Finally, during cold winters, burning of oil and wood for household heating can lead to high concentrations of PM_2.5_.

A use case is related to street cleaning, as the road owners in the area (Trondheim municipality, Trøndelag county and the Norwegian Road Authorities) are responsible for avoiding unhealthy levels of particle dust. Their available cleaning actions include washing, brushing and spraying and are carried out by special vehicles. In the past, a regular schedule for cleaning was applied but this has been changed to avoid unnecessary actions and currently cleaning is based on a subjective anticipation of needs, i.e., by foreseeing high pollution levels. This problem is challenging and has so far been solved by operators of the services using their experience to assess the current situation and trends and “having a quick look at the weather forecast”. Recently, the contract for cleaning actions have been put out for private offers and thus the experiences of operators might vary as new contracts are made.

The use of early warning systems for public health is cited as an important use case for pollution measurements [10]. However, this is an example of a wider range of important applications, apart from the national and international regulations that countries must follow, which arise from pollution-sensing networks. Asthmatic people living in crowded cities, parents deciding in which neighborhood to raise their children, or policy makers in public authorities, are examples of important target groups that need to consider air quality levels in their decision making, either in their daily lives or in strategic planning. For instance, asthmatic people might want to be notified if there is a prediction of high pollution levels in the next day, parents might decide where to buy a house depending on the average pollution at a location or policy makers might have to decide whether to restrict traffic at certain areas or periods. Therefore, there are groups that benefit from data outputs from air quality sensor networks.

In the concrete case of our pilot, discussions with Trondheim municipality resulted in the development of several scenarios in which air quality data can be used by the municipality itself, but also allows third parties to create novel services. One starting point for the municipality was the need to have good quality data with the final objective of making it available to the general public. Furthermore, within the wide range of possibilities for application that use air quality data, the municipality showed a particular interest in scenarios involving: (i) the provision of warnings for increasing pollution levels to operators of cleaning services; (ii) decision-making support in the form of visualizations of measured and forecasted air quality levels for the decision-makers at the municipality; (iii) monitoring and forecasting services delivered through mobile apps for citizens to plan their activities (short term) and to incentivize them to choose green transportation options; (iv) running what-if analyses to gain knowledge on the effects that regulating traffic patterns will have on pollution levels. In Section 4 we will provide more details regarding the first option.

### 2.3. Low-Cost Sensor Network in Trondheim

**Figure 2:**
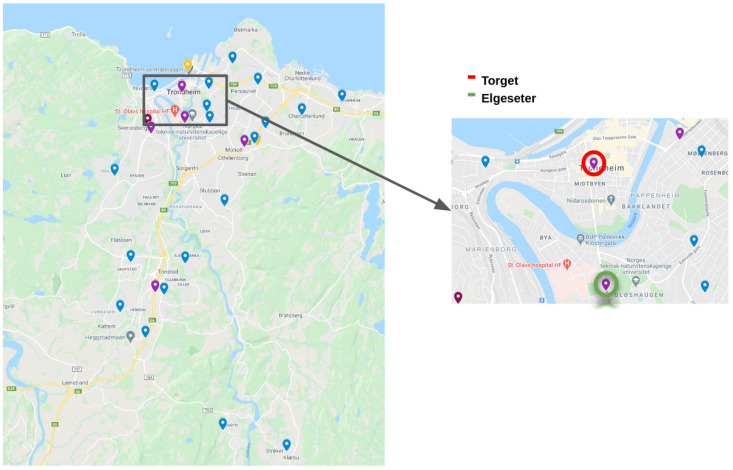
(**Left**): sensor network map (**Blue**: deployed low-cost sensors; **Yellow**: planned low-cost sensors; **Purple**: NEA network of industrial sensors). (**Right**): highlight of locations where low-cost sensors are co-located with reference sensors.

**Figure 3:**
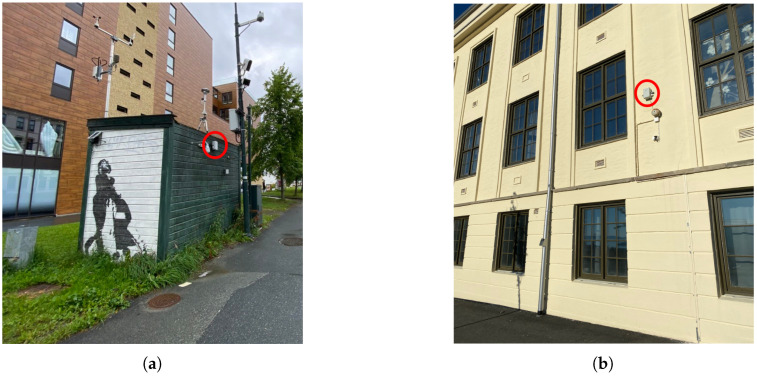
Photographs of the placement of low-cost sensors, highlighted in the pictures. (**a**): Co-located with a reference sensor at *Elgeseter*; (**b**): at *Berg* school.

The new network consists of a total of 25 new sensors, with 23 already deployed and 2 still to be placed. The already deployed sensors have been placed in strategic places, mostly schools and kindergartens, in Trondheim. This choice was made by the municipality considering three factors (More info at https://sites.google.com/trondheim.kommune.no/kunnskapsdeling/luftkvalitet-engelsk, accessed on 3 May 2021): first, those are owned by the municipality and, therefore, there are fewer logistic restrictions on the installation of sensors; second, the users of such places are groups of particular risk (for instance, children); third, their relative location and lack of data for a given area. Future deployment plans include mobile sensors, to be positioned on top of urban buses. From all the low-cost sensors, two are co-located (In the context of this paper, we define co-location as the placement of low-sensor devices and industrial sensors at Elgeseter and Torvet stations as close as possible due to practical considerations. The distance between air intakes is less than 2 m and, in the case of sensors in high traffic sites, both are facing the main road.) with reference air quality stations (*Elgeseter* and *Torget*), which are part of a national network of industrial sensors, in order to be able to obtain ground truth data for analysis of the data. In Trondheim’s area there are five industrial sensors, owned either by the municipality or the road administration, and the Norwegian Institute for Air Research classifies them as either traffic or background stations. Under this classification, *Torget* is a background station while *Elgeseter* and all other sensors are traffic stations. Figure 2 shows the locations of the sensors in the network, with a highlight on the locations where low-cost sensors are co-located with reference sensors, while Figure 3a shows a photograph of the setup at *Elgeseter*, with a low-cost sensor mounted on a reference sensor.

**Figure 4:**
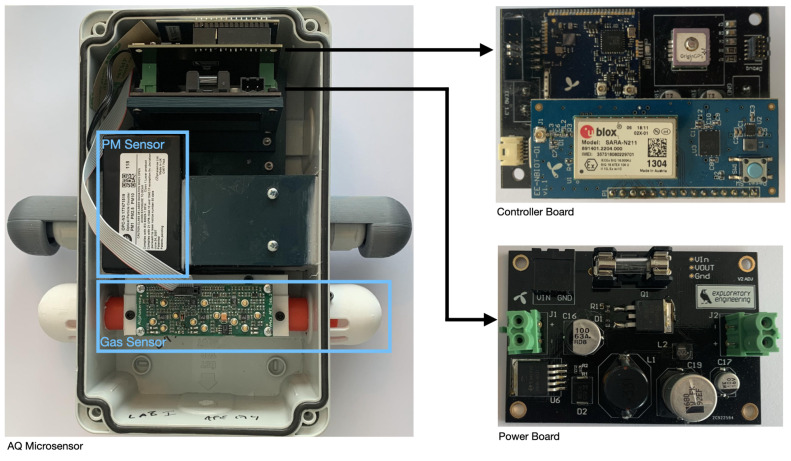
Photo of deployed sensor casing, including gas and particulate matter sensors, controller and power boards, and respective legend.

All sensors were installed with appropriate casing, as shown in Figure 4. The sensor board was designed with the ability to detect chemical pollutants (nitric oxide (NO), nitrogen oxide (NO_2_) and ozone (O_3_)) and particulate matter (PM_1_, PM_2.5_ and PM_10_). For gas sensing, the following Alphasense sensors are used: NO-A4 for NO [11], NO2-A43F for NO_2_ [12] and OX-A431 for O_3_ [13], with an Alphasense 3-sensor AFE board support circuit [14], while PM is measured with an Alphasense OPC-N3 [15]. Each board includes an Amphenol Chipcap 2 [16] temperature and humidity sensor, and a GPS module with an OriginGPS Hornet ORG1510-MK05 [17]. In this paper we will deal with PM measurements; therefore we detail in Table 1 the technical specification of the Alphasense OPC-N3 sensor, while we refer for the respective datasheets for all other components.

The global cost of deployment for this low-cost network is estimated to be around NOK (Norwegian kroner) 10,000–20,000 (∼EUR 1000–2000) for components and installation, per device. For the industrial reference sensors the estimated cost is around NOK 400,000–800,000 (∼EUR 40,000–80,000) per device, making it around 50 times more expensive than the low-cost sensors. The operating infrastructure cost is similar for both kinds of sensors. Our low-cost sensor network communicates through NB-IoT, with a yearly subscription costing 100 NOK (∼EUR 10) per device. Each sensor communicates independently to the server and 4G and 5G networks have been carefully designed to be able to handle massive numbers of IoT devices communicating over the NB-IoT protocol. It should also be noted that the operational costs for industrial reference sensors are substantial as these sensors are subject to strict routines of maintenance and calibration, to ensure high quality data.

A general challenge with chemical sensors is stability, since the performance and sensitivity degrade over time, which in turn limits the operating time before re-calibration is required [18], but calibration procedures with machine learning methods are also possible [19]. Nonetheless, in this paper we focus on particulate matter (PM) detection, as it is the pollutant that is less out of the control of the local municipality. The OPC-N3 sensor measures particle counts in 24 bins from 0.35 to 40 m by illuminating one particle at a time with a laser, and measuring the intensity of scattered light. The amount of scattered light is a function of the particle size, which is calibrated using a proprietary algorithm from Alphasense. A new setting (increasing the time the fan in the devices is on for each measurement) might result in somewhat better correlation with the reference sensors (at Torvet and Elgesetergate). This setting has recently also been applied to the rest of the sensors. There are interesting lessons that can be learned from this case regarding challenges encountered when putting IoT sensors to work in harsh conditions; among others, covers used to prevent humidity and salt from reaching the electronics also pose a problem to obtain the needed airflow. This can be solved by spending more power (longer fan-on time), which again poses a new problem if the device does not have a fixed power connection. Another example of challenges in harsh conditions is the need to put the sensors out of reach of people and thereby in sub-optimal locations for the measurements of air quality (Figure 3b), as there can be huge variations from the street level to high up on a building wall.

The deployment of the low-cost sensor network was finalized in late June 2020, with data collection starting at that point. Initial analysis of data incoming from the reference locations showed that the deployed sensors underestimated the pollution levels, with a weak correlation between reference sensors. The OPC-N3 sensor has some tunable parameters, with the most important for data quality being the sampling interval. After deployment several configurations were tested in one sensor, with the final configuration being set to a sampling interval of 1 min. The whole network was configured with the same parameter configuration on 12 November 2020. Therefore, throughout this paper we will limit our analysis to data received in the period between 15 November 2020 and 23 February 2021, with data being sent continuously 24 h per day and seven days per week. For the sake of preparing the dataset for the several components of this paper we selected this cutoff date, but the network is continuously up and running.

**Figure 5:**
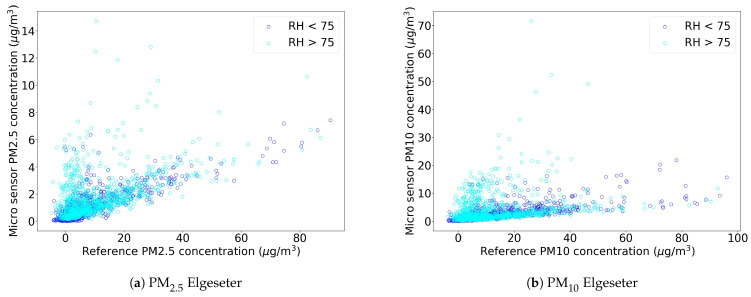
Scatter plots between low-cost sensor data and references with data collected between 15 November 2020 and 23 February 2021 at *Elgeseter*.

Previous studies on the quality of Alphasense low-cost sensors, both in laboratory conditions [20] and outdoors [21,22], reported that they tend to overestimate the real PM concentration with a larger number of outliers under high humidity conditions [21,22]. Furthermore, the influence of meteorological conditions was shown to be important for different types of low-cost sensors [23,24]. The measurements from our network showed a behavior with a large underestimation of pollution levels. Table 2 shows the summary statistics for the pollutant datasets, both from low-cost sensors and their respective references, together with correlations between low-cost and reference sensors. Figure 5 shows the analysis on our data, comparing PM_2.5_ and PM_10_ measurements from a low-cost sensor and a reference sensor, colored according to the relative humidity. In accordance to the cited literature, we observe that low-cost sensors measurements are affected by external factors, such as humidity and possibly others meteorological factors (temperature, air pressure, etc.). This, together with a low correlation between low-cost and reference sensors, led us into investigating automatic calibration models that take external features into account.

## 3. Data Calibration

### 3.1. Datasets

Due to the deployment process previously described, we had available low-cost sensor data from 15 November 2020 to 23 February 2021. The sensors are connected via Narrowband IoT to an air quality server backend, which provides data to end users as a SQLite database. For the moment, these are not yet publicly available data. As for the reference sensors, data are fetched through a public API (https://api.nilu.no/, accessed on 3 May 2021) of the Norwegian Institute for Air Research (NILU). For a fair comparison, data from low-cost sensors are aggregated with hourly averages, to be in the same format as the available data for reference sensors.

As additional inputs we use weather and traffic data. As mentioned, it was shown in field evaluation studies that the performance of low-cost sensors is influenced by weather and, in particular, humidity was typically mentioned. We have two sources of weather data. First, the Norwegian Meteorological Institute has a public API (https://frost.met.no/index.html, accessed on 3 May 2021) with historical and real time information about weather measurements from different stations across the country. Several are available in Trondheim but, unfortunately, most do not have available measurements for all of the most important weather elements. Therefore, we chose to use data from a single station (Voll), which are the most complete, as representative of the weather conditions; the station is located less than 5 km from the reference locations. Data from this source consist of temperature, relative humidity, precipitation, air pressure, wind speed and wind direction. Additionally, the OPC-N3 sensor also provides measurements of temperature and humidity, which might be much more useful as local measurements. These measurements are, then, taken inside the box where sensors and boards are kept, which means that their magnitudes are not comparable with temperatures measured outside. Nonetheless, we observed a good correlation between these measurements and the ones from the Meteorological Institute (0.96 for both low-cost sensors), indicating that we might rely on them for remotely located sensors. However, the rate of faulty measurements are also higher for those, particularly the temperature reading with a rate of faulty measurements that can go up to 25%. We will elaborate on this and test in practice what the difference is when presenting results for automatic calibration models.

For traffic, the Norwegian Public Roads Administration hosts a variety of induction loop sensors located at relevant entry points of the city of Trondheim. The sample rate is on a per hour basis and counts how many vehicles passed through the sensors. These data are available through a public API (https://www.vegvesen.no/trafikkdata/start/, accessed on 3 May 2021), and we include in our dataset traffic counts from the three traffic sensors closest to the reference sensors.

### 3.2. Single Sensor Calibration

#### 3.2.1. Method

Nonetheless, training of models for correction of low-cost sensor data against a reference has proven to produce good results [25]. To test the accuracy of such approaches in our setup we trained a random forest regressor with a different number of input features, in both locations with a reference sensor. Random forests showed good results in the calibration of other types of low-cost sensors [25,26], and thus we selected it as the method for the calibration procedure. In this approach, the input is the measurement of the low-cost sensor and an additional number of context features, and the target is the measurement from the reference sensor. We used the *Elgeseter* sensor for training and initial testing, and then also tested the trained calibration model with data from the *Torget* sensor. For all sensors we evaluated the performance with the root mean squared error (RMSE) and the correlation coefficient (R^2^) metrics.

Additionally, we tested a different approach that attempts to classify the current real pollution level, instead of predicting the exact value. This approach is based on the observation that, often, one might not be interested in the exact value but rather on the air quality level in the surroundings of a sensor. For instance, this could be used as a means to build target datasets for warning systems or to provide a more structured information to end users, through map visualizations in mobile apps. Therefore, we trained a random forest classifier with the same setup as described for the regressor. As performance metrics we evaluated recall, precision and area under the receiver operating characteristic curve (AUC).

Finally, we calibrated the whole dataset for both sensors (low-cost sensors at Torget and Elgeseter) and recalculated the correlation against the reference sensor. In this case, we used the model tested with data from the Elgeseter sensor and used the same model to calibrated data from the Torget sensor.

#### 3.2.2. Results

Table 3a shows the results obtained for sensor calibration with regression. We note that the calibration model tended to overfit when using only pollutant data as input, with a good training score but poor testing scores. This can be explained by the limited amount of data, combined with pollutant data not being enough for the algorithm to find calibration patterns. On the other hand, test scores improved as we included more input features and, in particular, using weather and all available measures for different particulate matter sizes seemed to make most of the difference. In relation to weather features we tested different combinations as input to the model, using both meteorological data from the Meteorological Institute and data obtained by the OPC-N3 sensor, which measures local temperature and humidity. As mentioned, using weather features significantly improved the model, with better results when using official weather data when compared to OPC-N3 weather data. While official weather data are measured in a different location in the city, those are more accurate than OPC-N3 measurements. Moreover, the high rate of faulty measurements for OPC-N3 temperature highly influenced the training process, especially in the presence of limited training data.

In turn, Table 3b presents results for the calibration procedure with a classification model, i.e., with the goal of assigning air quality levels to the low-cost sensor data. Also in this case, the influence of weather features on the model performance is noticeable, although using all pollutant measures did not seem to have such an influence as in the regression model. Moreover, classification of PM_2.5_ levels resulted in better performance than for PM_10_. Particularly, the model for PM_10_ seems to have had difficulties in maintaining the balance between false positives and negatives, as we can see by looking into the recall and precision values; only when combining PM_10_ and weather inputs are both metrics above 0.5.

Generally, the calibration procedure output better results for PM_2.5_ than PM_10_. For the regression model this can be partially explained by the fact that PM_2.5_ values are lower than PM_10_, therefore yielding a lower RMSE. However, even the calibration model, which should not be dependent on the magnitude of the time series, obtained better performance with PM_2.5_. This result comes in line with the observation that the initial correlation between the low-cost and reference sensors for PM_10_ was worst than for PM_2.5_, which indicates worse data quality for this measurement. Additionally, evaluation studies showed PM_10_ measurements with OPC sensors to have a higher variance and be more affected by outliers.

**Figure 6:**
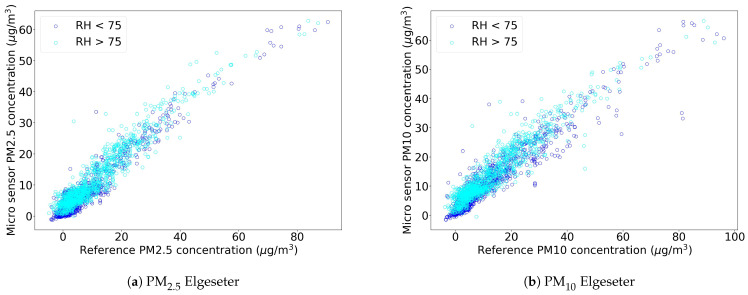
Scatter plots between calibrated low-cost sensor data and references. Data collected between 15 November 2020 and 23 February 2021 at *Elgeseter*.

After analyzing the performance of calibration models, we proceeded to calibrated the whole dataset with the trained model. Table 4 shows an updated analysis on data statistics, but now with the calibrated low-cost sensor dataset. The calibration model used is the one with inputs PM_1_, PM_2.5_, PM_10_ and weather. This shows the effectiveness of the calibration procedure, with a significant increase in the correlation scores. It is particularly interesting to note that the correlation in the *Torget* sensor also significantly improved even though the model was trained on data from *Elgeseter* and then transferred. Moreover, the effect of external features, such as humidity, was reduced by the calibration procedure as seen in Figure 6. Here, we replicated the analysis of Figure 5 using calibrated data. The magnitude of the measurements from low-cost sensors are now much more in line with the references.

### 3.3. Hyperlocal Prediction of Air Pollutants

#### 3.3.1. Method

In this method, we tested the feasibility of extrapolating pollutant values for different locations in the Trondheim area from the available low-cost sensor data. We trained a random forest regression model that takes as inputs the pollutant values of each low-cost sensor and predicts PM_2.5_ and PM_10_ concentrations for a target location. The available industrial sensors provide us with accurate measurements of pollutants for five different geographical locations that are used as ground truth data. The network of sensors used in our model consists of 23 low-cost sensors and 5 reference sensors whose geographical location can be seen in Figure 2. Apart from the main pollutants, low-cost sensors also detect humidity and temperature levels, allowing us to model cross-sensitivities between measurements and weather conditions. To account for the relative distance between low-cost sensors and target sensors, we calculated the Haversine distance of every low-cost sensor from the target and added it as a feature in the dataset.

Meteorological and traffic data of the area were available but they were not included in the dataset for this testing scenario. Our motivation was to test if the network of low-cost sensors is sufficient to achieve good results without the use of external data sources. Since meteorological stations and traffic sensors are limited to a few specific locations, we would have to limit our testing to those areas in order to avoid added bias in favor of low-cost sensors located next to them, compared to low-cost sensors that are further away. Moreover, the calibration process of low-cost sensors can assist in minimizing the interference of unknown variables with our measurements.

**Figure 7:**
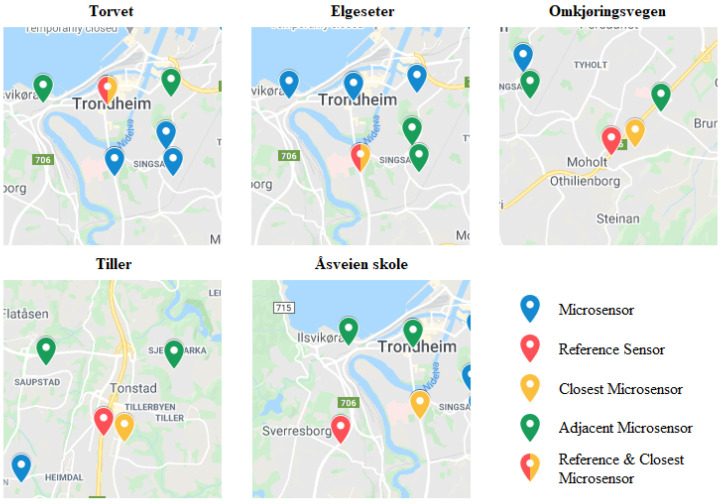
Adjacent low-cost sensors used in the baseline models for every reference sensor. **Red**: reference sensors. **Green**: two of the three closest adjacent low-cost sensors used in the average value baseline model. **Yellow**: the third and closest adjacent low-cost sensor used both in the average value and closest low-cost sensor baseline models. **Red/Yellow**: in the Torget and Elgeseter areas there is a low-cost sensor next to the reference sensor, so a dual coloring is used to mark both.

Various regression algorithms, such as Support Vector Regressor, XGBoost, and Random Forest Regressor, were evaluated during the testing phase, with Random Forest Regressor returning slightly better results. An additional advantage of the random forest algorithm is its ability to calculate the most important features of the dataset that, in turn, assist in the interpretation of the predictions, detection of high-impact areas of pollution, and comparison of cross-correlations between air pollutants. The average value of the three closest low-cost sensors and the value of the closest low-cost sensor are used as a baseline of performance for our model predictions. Visual estimation of the proximity of adjacent low-cost sensors to the reference sensors is provided in Figure 7.

#### 3.3.2. Results

The test set results of our model are shown in Table 5. For both pollutants, the regression model achieved a low root mean squared error (RMSE) and a high correlation coefficient (R^2^) compared to the baseline values. Specifically, the negative R^2^ value of the closest sensor to the target highlights the extreme fluctuations of pollutant concentration based on external factors, when we account for distance. By contrast, the model achieved R^2^ values higher than 0.6 for both pollutants, capturing cross-correlations among the features sufficiently.

There are certain limitations in the model that have to be taken into account. There were only four months of low-cost sensors data since the last calibration in November, so they do not contain enough information for seasonality and yearly trends. Moreover, the five reference sensors cover a small part of the Trondheim area, offering limited ground truth data that, in turn, affects the generalization of the model. Additional online time of low-cost sensors and further improvements in their calibration will allow for a better model to be trained in the future.

## 4. Applications

### 4.1. Warning System for Increased Particle Dust Levels

On the background described in Section 2, the environmental unit in Trondheim municipality supported the idea of exploring the possible development of a warning system for increased particle dust levels based on historical data including pollutants, weather and traffic. The main users of the system would be the operators of the cleaning services, but also people at the environmental unit of the municipality might want to use the system for follow-up of contracts in their role as pollution authorities. The initial requirements for the system (identified in collaboration with the municipality) were to: 1. Offer warnings for situations with high levels of particle dust, 24 h ahead of time; 2. Describe the quality of the warnings to the users in an understandable format; 3. Provide an indication of on which features the warnings have been based.

We address this problem as a classification problem with two classes based on the characteristics of a state, i.e., selected features describing the current situation:

- Class 1: States that are followed by high pollution levels (i.e., above a given threshold) for at least one of the 24 following hours.
- Class 2: States that are not followed by high pollution levels.

Then, if a state is identified as Class 1 a warning will be issued to the operators of the street cleaning service.

Calibrated data might be beneficial for the success of the application. First, as previously mentioned, low-cost sensor data correlate better with a reference sensor after calibration. Moreover, if no reference sensors are available close to low-cost sensors then calibrating their data and developing warning systems based on them might be the only available option. Therefore, we tested a warning system in a location with both a low-cost and reference sensor, comparing two different models that receive as input data from each sensor seperately. As performance metrics, we used the recall, precision and area under the receiver operating characteristic curve (AUC). Table 6 shows the results for the test set. Again, we had limited data available due to the deployment process of the low-cost sensor network, but they provided insight into the feasibility of using low-cost sensor data for this kind of warning system when comparing with reference data. We note that the behavior of the models was similar when using both sources of pollution data. The warning for PM_2.5_ seemed to be able to find more positive Class 1 samples than with PM_10_. Given the limited period of data available and the unbalanced nature of such datasets, further tests and data might be needed before deciding on a deployment of such models, but we believe that this indicates that using low-cost sensor data for warning systems in different locations in the city is feasible.

### 4.2. Visualizations

We used a number of visual analytics techniques to present the low-cost sensor data to the viewer, to allow for a deeper understanding of the data and the possible discovery of patterns. We have focused on presenting the spatio-temporal distribution of all types of collected measurements and the pollution associations between geographical areas.

**Figure 8:**
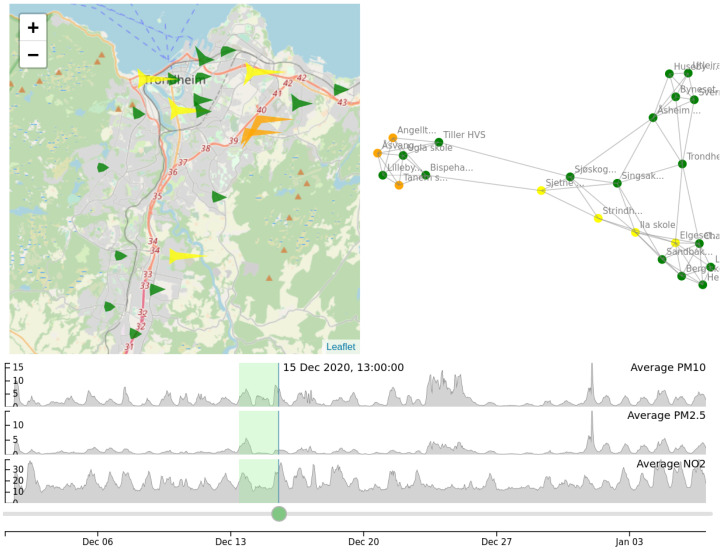
Visualizations for the low-cost sensor data. Left: map view; a star-like glyph is used to display the measurements of each sensor. Right: graph view; each sensor corresponds to a node, with nearby nodes denoting similar measurements. Bottom: timeline view; the progress of the pollutants in time.

**Figure 9:**
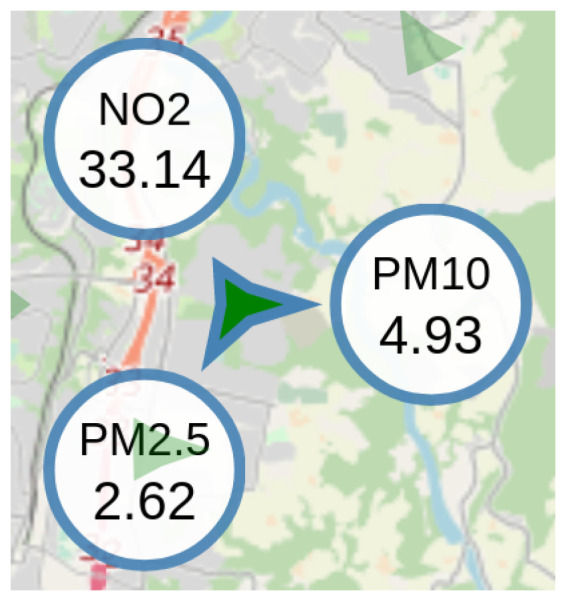
Close-up of a star glyph used in the map view. Each spike angle corresponds to a different type of measurement. The larger the spike’s length, the larger the corresponding measurement. The numeric details as shown here appear upon mousing over a selected glyph.

In order to show the spatio-temporal distribution of the collected measurements, we used a map view, locating all the sensors using their geographical coordinates, as can be seen in the left panel of Figure 8. In order to show as much information as possible in the map, we used star-like glyphs to display the information collected by each sensor at a specific time instance. Glyphs in general are compact representations that visualize multi-dimensional data by mapping multiple dimensions on multiple characteristics of a shape, such as a polygon, an arrow, etc. [27]. In this paper, we used star-like glyphs, which are a form of polygon for representing several attributes at once [28]. The distance of each star tip to the center of the star is proportional to the value for a specific type of measurement. In the example shown in Figure 8, three types of measurement are shown, namely PM_10_, PM_2.5_ and NO_2_. Each type of measurement corresponds to a specific angle around the center of the star glyph, as shown in Figure 9. The overall shape of the glyph is therefore characteristic of the vector of measurements collected by each sensor, allowing comparisons among sensors and geographical areas. The specific values of each measurement can be viewed by mousing over each glyph. The color of the glyphs shows the level of pollution (green: low levels; yellow: medium levels; orange: high levels; purple: very high levels—relative to the extent of the overall measurements, and adhering to the code used in the official air pollution forecasting service offered by NEA).

The timeline and slider at the bottom of the map allow the user to explore different time instances. Moving the slider back and forth, the user can view how the shapes of the glyphs change with time. This type of interaction allows temporal patterns or peculiarities to be detected by the user, along with the spatial patterns on the map. Seasonal patterns such as fluctuations in pollutants between day and night, as well as “waves” of pollution moving from south to north, can be seen using this kind of interaction.

Associations between the sensors are visualized using a graph-based view, as shown in the right panel of Figure 8. Graphs have been particularly suited in the literature for visualizing associations between entities [29]. Here, each node of the graph corresponds to a sensor. The graph is a *k*-nearest neighbor graph among the sensors. An edge between two sensors is placed if the two sensors are similar with respect to their history of measurements over a specified time window (indicated by a green shade at the timeline view). This type of visualization provides another map for the sensors, this time in the feature space, rather than the geographic space. As the history of measurements changes with time, so does the association between nodes and the overall structure of the graph. This can be seen interactively by using the same slider used for the map view. The user can further select groups of nodes on the graph to highlight the corresponding points on the map, as shown in Figure 8.

**Figure 10:**
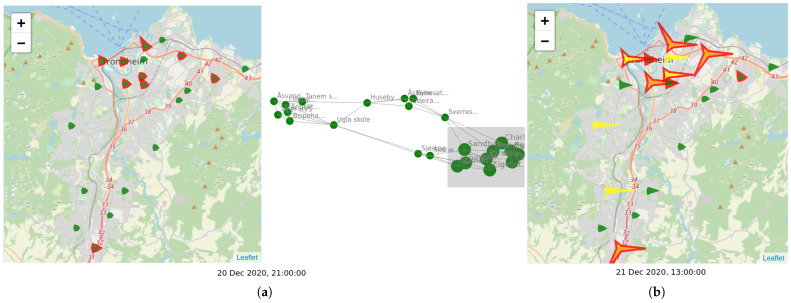
Example of linked graph and map visualization. (**a**) On a particular day, the graph view reveals groups of sensors with similar previous behavior. One group was selected by the user and the corresponding sensors are highlighted on the map. (**b**) A few hours later, the selected sensors exhibit similar behavior, measuring high pollutant concentrations, distinguished from the behavior of the other sensors.

The combined map and graph views can potentially reveal interesting patterns. An example is shown in Figure 10. Figure 10a depicts a particular snapshot in time, where the sizes and shapes of the sensor glyphs indicate low levels of pollution. The graph view for this instance reveals some groups of similar sensors located in the feature space. These groups have been formed by taking into account not only the measurements of this instance but also measurements of previous time instances within a short time window. In this example, the user has selected the rightmost group of graph nodes, and the corresponding sensors are highlighted in red in the map view. Most of the sensors are also located in nearby locations on the map, showing that feature similarity is relevant to geographic proximity. However, the group also contains a sensor at the far south, which is similar to the others only in the feature space, i.e., in its temporal behavior. Figure 10b shows the snapshot taken a few hours later. The previously selected sensors seem to behave similarly, measuring high pollution concentrations, in contrast to the non-selected ones, the majority of which stay at low levels. This change in behavior is also reflected in the far sensor to the south, which also exhibits the same behavior. This example shows that the sensor clustering at the feature space can reveal associations between sensors that go beyond geographic proximity, and that can be exploited for understanding pollution patterns and making predictions.

## 5. Conclusions

This paper discusses the challenges towards the development of innovative applications and services to improve air quality monitoring and decision-support for the municipality of Trondheim, in Norway. Air pollution is a highly local phenomena but monitoring it can be quite expensive with costs including, for example, air quality sensors, calibration procedures or measurement communication. To face this challenge a network of low-cost sensors was deployed throughout the city, to complement the (much smaller) network of industrial sensors which were previously available. Low-cost sensors drastically reduce the cost of deploying such networks but at the expense of noisier and often faulty measurements and, often, additional processing is needed to ensure good quality data further down in the service pipeline.

In our deployment we found the behavior of low-cost sensors to differ from the field studies publicly available. Contrary to those, our sensors tended to underestimate the pollution levels, when comparing against industrial reference sensors, at places where both sensor types are co-located. However, we showed how procedures based on machine learning have the potential to calibrate data incoming from low-cost sensors, with a high increase in post-calibration correlation against references, and captures the effects from external features such as humidity and other meteorological influences in the sensors’ output.

Calibrating pollution data is not an end in itself, but a means to produce meaningful inputs to services that can be used by the municipality, and citizens in general, for decision-making support. We illustrated two applications that benefit from the calibration procedure and showed how they can fulfill the desire for real-time services from the municipality: a warning system for prediction of pollution peaks in the next 24 h, and visualization techniques with spatial and temporal patterns of the measurements.

In the future we plan to continue working with the municipality to develop new services and functionalities that will be of interest to decision makers and the general public. In particular, initial steps were taken towards the development of a mobile app that can use data from the sensor network, combined with some of the visualization techniques here presented, to raise awareness among citizens and promote healthier lifestyles. Additionally, there is work towards the development of a digital twin of the city that incorporates a traffic simulation based on real data along the inclusion of several other emission sources to allow the generation of what-if analyses, i.e., test the effect of the municipality’s decisions through the simulation of future scenarios in a digital copy of the city, with traffic and pollution patterns modeling based on real measures [30]. All services are to be deployed through the European AI on-demand platform, currently under development under the AI4EU project. In relation to sensor calibration, we would like to test automatic transfer of calibration models through the whole sensor network. In this paper we show that transferring calibration models between sensors is feasible but the evaluation of such a procedure needs a reference sensor as a target. A possibility is to co-locate all sensors with references, gather data, calibrate them and then deploy to their final locations, but a more automatic solution would ease and speed the deployment process. Additionally, sensor placement methods [31] can be investigated in order to optimize the number of sensors needed to cover the map and the location to where deploy such sensors.

## Figures and Tables

**Table 1:** Technical specifications of Alphasense OPC-N3 particle monitor, as specified by the manufacturer [15].

Name	Measures	Detection Range (μm)	Num. Bins	Max Particle Count Rate	Max Coincidence Probability
Alphasense OPC-N3	PM_1_ PM_2.5_ PM_10_	0.35–40	24	10,000	0.84% at $10^{6}$ particles/L

**Table 2:** Data collected between 15 November 2020 and 23 February 2021 for reference and low-cost sensors at locations where both are co-located. Data were not pre-processed and we used reference data “as is” from public API; therefore we maintained negative values that might be explained by the calibration procedures for industrial reference sensors.

(**a**) Data statistics. The count field is the total number of valid readings, while the other fields are in μg/m^3^.								
	**Elgeseter**				**Torget**			
	**Reference**		**Low-Cost**		**Reference**		**Low-Cost**	
	PM_2.5_	PM_10_	PM_2.5_	PM_10_	PM_2.5_	PM_10_	PM_2.5_	PM_10_
**count**	2314	2314	2344	2344	2340	2340	2345	2345
**mean**	9.09	12.88	1.43	3.26	9.06	11.45	1.11	2.00
**std**	11.73	13.67	1.46	4.28	10.64	12.57	1.02	1.86
**min**	−4.81	−3.80	0.00	0.00	0.10	0.10	0.02	0.04
**Q1**	1.86	3.84	0.45	0.97	2.50	3.10	0.39	0.72
**median**	4.92	8.17	0.98	1.94	5.00	6.70	0.82	1.45
**Q3**	12.16	17.94	1.87	3.73	11.30	15.00	1.50	2.64
**max**	90.44	96.10	14.70	71.48	83.40	135.10	10.33	13.72
(**b**) Pearson correlation (*r*), slope and intercept between low-cost sensor and reference.								
**Sensor Location**			**Particle Size**		r		**Slope + Intercept**	
Elgeseter			PM_2.5_		0.61		0.07+0.73	
			PM_10_		0.36		0.11+1.73	
Torget			PM_2.5_		0.68		0.07+0.47	
			PM_10_		0.42		0.07+1.20	

**Table 3:** Calibration results with both prediction and classification models.

(**a**) Calibration with a random forest regressor. Data collected between 15 November 2020 and 23 February 2021. Train–test split: 0.75/0.25.						
**Inputs**	**Training**		**Test (Elgeseter)**		**Test (Torget)**	
	**RMSE**	**R^2^**	**RMSE**	**R^2^**	**RMSE**	**R^2^**
**Elgeseter, PM_2.5_**						
PM_2.5_	6.28	0.76	8.57	−0.13	8.32	0.39
PM_2.5_, Temperature (OPC), Humidity (OPC)	2.16	0.97	6.59	0.32	5.71	0.64
PM_2.5_, Temperature, Humidity	2.89	0.95	5.09	0.60	5.43	0.74
PM_2.5_, Weather	2.48	0.96	4.66	0.67	4.71	0.80
PM_1_, PM_2.5_, PM_10_, Weather	1.83	0.98	3.77	0.78	4.05	0.86
PM_1_, PM_2.5_, PM_10_, Weather, Traffic	1.79	0.98	3.66	0.79	4.03	0.86
PM_1_, PM_2.5_, PM_10_, Weather, Hour of Day, Day of week	1.9	0.98	3.6	0.8	4.3	0.83
**Elgeseter, PM_10_**						
PM_10_	8.91	0.62	9.49	0.22	10.99	0.20
PM_10_, Temperature (OPC), Humidity (OPC)	3.51	0.94	9.94	0.21	7.91	0.52
PM_10_, Temperature, Humidity	4.75	0.89	7.27	0.54	7.45	0.63
PM_10_, Weather	3.84	0.93	5.83	0.71	6.37	0.73
PM_1_, PM_2.5_, PM_10_, Weather	2.49	0.97	5.74	0.72	5.34	0.81
PM_1_, PM_2.5_, PM_10_, Weather, Traffic	2.41	0.97	5.38	0.75	5.33	0.81
PM_1_, PM_2.5_, PM_10_, Weather, Hour of Day, Day of week	2.43	0.97	5.30	0.76	5.32	0.81
(**b**) Calibration with a random forest classifier. Data collected between 15 November 2020 and 23 February 2021. Threshold of $20\mu g/m^{3}$ for PM_2.5_ and $30\mu g/m^{3}$ for PM_10_. Train–test split: 0.75/0.25.						
**Inputs**	**Test (Elgeseter)**			**Test (Torget)**		
	**Recall**	**Precision**	**AUC**	**Recall**	**Precision**	**AUC**
**Elgeseter, PM_2.5_**						
PM_2.5_	0.39	0.47	0.85	0.42	0.38	0.78
PM_2.5_, Temperature (OPC), Humidity (OPC)	0.48	0.62	0.94	0.54	0.66	0.9
PM_2.5_, Temperature, Humidity	0.63	0.64	0.93	0.56	0.65	0.9
PM_2.5_, Weather	0.69	0.79	0.96	0.63	0.78	0.92
PM_1_, PM_2.5_, PM_10_, Weather	0.76	0.74	0.97	0.66	0.78	0.94
PM_1_, PM_2.5_, PM_10_, Weather, Traffic	0.8	0.8	0.98	0.63	0.79	0.92
PM_1_, PM_2.5_, PM_10_, Weather, Hour of Day, Day of week	0.73	0.78	0.97	0.64	0.79	0.93
**Elgeseter, PM_10_**						
PM_10_	0.33	0.28	0.75	0.18	0.18	0.68
PM_10_, Temperature (OPC), Humidity (OPC)	0.61	0.47	0.91	0.29	0.49	0.79
PM_10_, Temperature, Humidity	0.33	0.32	0.93	0.37	0.55	0.84
PM_10_, Weather	0.55	0.75	0.95	0.46	0.66	0.85
PM_1_, PM_2.5_, PM_10_, Weather	0.61	0.61	0.97	0.47	0.77	0.89
PM_1_, PM_2.5_, PM_10_, Weather, Traffic	0.48	0.62	0.96	0.46	0.74	0.88
PMPM_1_, PM_2.5_, PM_10_, Weather, Hour of Day, Day of week	0.48	0.62	0.97	0.44	0.72	0.88

**Table 4:** Data collected between 15 November 2020 and 23 February 2021 for reference and calibrated low-cost sensors at locations where both are co-located (in μg/m^3^). Both sensors were calibrated using the model trained with Elgeseter data. Data were not pre-processed and we used reference data “as is” from the public API; therefore we maintained negative values that might be explained by the calibration procedures for industrial reference sensors.

(**a**) Data statistics. The count field is the total number of valid readings, while the other fields are in μg/m^3^.								
	**Elgeseter**				**Torget**			
	**Reference**		**Low-Cost**		**Reference**		**Low-Cost**	
	PM_2.5_	PM_10_	PM${}_{2.5}^{calib}$	PM${}_{10}^{calib}$	PM_2.5_	PM_10_	PM${}_{2.5}^{calib}$	PM${}_{10}^{calib}$
**count**	2314	2314	2344	2344	2340	2340	2345	2345
**mean**	9.09	12.88	9.63	12.95	9.06	11.45	8.66	10.96
**std**	11.73	13.67	9.86	10.45	10.64	12.57	9.00	9.32
**min**	−4.81	−3.80	−1.23	−1.54	0.10	0.10	−1.18	−1.33
**Q1**	1.86	3.84	3.52	6.12	2.50	3.10	3.11	5.27
**median**	4.92	8.17	6.33	9.54	5.00	6.70	5.83	8.12
**Q3**	12.16	17.94	12.10	17.48	11.30	15.00	9.92	14.05
**max**	90.44	96.10	62.79	66.59	83.40	135.10	62.73	65.46
(**b**) Pearson correlation (*r*), slope and intercept between calibrated low-cost sensors and reference.								
**Sensor Location**			**Particle Size**		r		**Slope + Intercept**	
Elgeseter			PM_2.5_		0.96		0.8+2.2	
			PM_10_		0.94		0.73+3.45	
Torget			PM_2.5_		0.91		0.78+1.47	
			PM_10_		0.88		0.68+3.13	

**Table 5:** Pollutants prediction based on low-cost sensor data. Data collected between 15 November 2020 and 23 February 2021. Train–test split:0.75/0.25.

Models	Training Set		Test Set	
	RMSE	R^2^	RMSE	R^2^
**PM_2.5_ Prediction**				
Random Forest Regressor	1.251	0.958	3.397	0.689
Closest low-cost sensor value	7.701	−0.581	7.509	−0.519
Closest 3 low-cost sensors avg value	7.516	−0.505	7.445	−0.493
**PM_10_ Prediction**				
Random Forest Regressor	1.982	0.951	5.229	0.668
Closest low-cost sensor value	25.324	−6.840	71.144	−62.049
Closest 3 low-cost sensors avg value	15.244	−1.841	29.943	−9.861

**Table 6:** Results for prediction of air quality levels with low-cost sensor data (calibrated) and NEA as input. Data collected between 15 November 2020 and 23 February 2021. Train–test split: 0.75/0.25. Thresholds: PM_2.5_ = 25, PM_10_ = 45.

Inputs	Test (w/Low-Cost Sensor)			Test (w/NEA)		
	Recall	Precision	AUC	Recall	Precision	AUC
**Elgeseter, PM_2.5_**						
Pollutants	0.69	0.36	0.65	0.5	0.44	0.66
Pollutants, Weather	0.75	0.41	0.68	0.73	0.44	0.68
Pollutants, Weather, Traffic	0.72	0.42	0.68	0.70	0.47	0.69
Pollutants, Weather, Traffic, Temporal	0.74	0.41	0.69	0.67	0.42	0.68
Pollutants, Weather, Traffic, Temporal, Delta	0.75	0.39	0.69	0.73	0.44	0.71
Pollutants, Weather, Traffic, Temporal, Delta, Forecast	0.78	0.44	0.72	0.80	0.48	0.73
**Elgeseter, PM_10_**						
Pollutants	0.30	0.21	0.67	0.28	0.31	0.61
Pollutants, Weather	0.37	0.3	0.83	0.39	0.31	0.84
Pollutants, Weather, Traffic	0.35	0.32	0.83	0.31	0.33	0.84
Pollutants, Weather, Traffic, Temporal	0.28	0.33	0.8	0.26	0.41	0.82
Pollutants, Weather, Traffic, Temporal, Delta	0.31	0.46	0.85	0.26	0.47	0.85
Pollutants, Weather, Traffic, Temporal, Delta, Forecast	0.37	0.59	0.83	0.30	0.55	0.81
